# Untreated Acute Posterior Multifocal Placoid Pigment Epitheliopathy (APMPPE): a case series

**DOI:** 10.1186/s12886-018-0744-z

**Published:** 2018-03-20

**Authors:** Olivia Xerri, Sawsen Salah, Dominique Monnet, Antoine P. Brézin

**Affiliations:** Department of Ophthalmology, Hôpital Cochin, Assistance Publique Hôpitaux de Paris, Université Paris Descartes, Paris, France

**Keywords:** Acute posterior multifocal placoid pigment epitheliopathy, Inflammatory disease, Posterior uveitis, Retina, Retinal pigment epithelium

## Abstract

**Background:**

Acute Posterior Multifocal Placoid Pigment Epitheliopathy (APMPPE) is a rare inflammatory eye disease that affects the Retinal Pigment Epithelium and outer retina. The purpose of this study was to describe its presentations, as well as its prognosis in a series of untreated patients.

**Methods:**

Records of patients seen in the department of Ophthalmology at Cochin University Hospital, Paris, between April 2002 and June 2015 were retrospectively studied. Patients were included if they presented with the typical findings of APMPPE characterized by whitish or yellowish bilateral placoid lesions, a typical pattern of early hypofluorescence and late hyperfluorescence on fluorescein angiography. Only untreated patients who had been followed for at least 1 month were included.

**Results:**

Out of 22 patients’ records with a diagnosis of APMPPE, 10 patients (9 women, 1 man), with a mean age of 24.5 ± 4.2 years, fulfilled the study criteria with a diagnosis of typical untreated APMPPE. Prodromal symptoms were reported in 7/10 patients. Macular lesions were observed in 18/20 eyes. Sub-retinal fluid was seen at presentation in 3 eyes. Initial mean BCVA was 0.56 ± 0.81 LogMAR [− 0.10 to 2.30]. In 9 out of 10 cases, the time interval between manifestations in the first affected eye and the fellow eye was less than 3 days. After 1 month, BCVA had improved to 0.05 ± 0.089 LogMAR [0–0.3], with a decimal BCVA ≥0.8 in 17/20 eyes.

**Conclusions:**

In these 10 cases of untreated APMPPE, a favorable outcome was observed.

## Background

Acute posterior multifocal placoid pigment epitheliopathy (APMPPE) is a rare inflammatory disease that typically affects healthy young adults. Five decades after its first description, it remains debated whether the primary tissue involved is the choriocapillaris or the retinal pigment epithelium [1–4]. Patients typically present with a rapid onset of visual loss associated with central and paracentral scotomas. The disease is usually bilateral with both eyes involved within a week, but may be asymmetrical [5, 6]. Flu-like symptoms often precede the onset of the disease, but central nervous system (CNS) involvement ranging from headaches to diffuse cerebral vasculitis is also observed [7]. Cases following immunizations or infections have also been reported [8].

The observation of the fundus typically shows multifocal, yellowish-white, placoid lesions, varying in size, located from the posterior pole to the mid-periphery. On fluorescein angiograms, the lesions show early hypofluorescence and late hyperfluorescence (“blocks early, stains late”). Indocyanine green angiography reveals early and late hypofluorescence. The lesions fade gradually within weeks, to be replaced by varying degrees of hyperpigmentation and sometimes by retinal pigment epithelium (RPE) atrophy.

Optical Coherence Tomography (OCT) imaging and especially spectral domain Optical Coherence Tomography (SD-OCT) allowed the description of an aspect of heterogeneous sub-retinal-fluid (SRF) at the very early stages of the disease, evolving to outer nuclear layer (ONL) hyperreflectivity before thinning [9, 10]. Prior to the complete healing, a phase of disruption of the inner segment/ outer segment (IS/OS) layer with hyperreflectivity of the RPE can last until the third month [9].

The natural history of APMPPE was initially described as globally favourable, [11, 12] but some reports show that patients may experience an incomplete visual recovery [13–15]. Hence, whether to treat patients with APMPPE or not remains debated. Some authors have advocated systematic corticosteroid treatment, other limit the indications of therapy to cases with macular involvement [10, 16–18]. To our knowledge, there are no recent series using modern imaging techniques focused on untreated APMPPE patients. The purpose of our study was to assess the ocular and extra-ocular features, as well as the visual prognosis in a group of untreated patients.

## Methods

This was a retrospective study of patients with a diagnosis of APMPPE seen between April 2002 and June 2015 in the department of Ophthalmology of the Cochin University Hospital, Paris. Patients were included if they demonstrated the typical fundus and fluorescein findings of APMPPE. Required features were multiple, geographic, deep white-yellowish lesions that were hypofluorescent at the early stages and hyperfluorescent at the late stages of the angiography. Only untreated patients with a follow-up of at least one-month were included. Patients with atypical features such as multifocal choroidal lesions and/or atrophic punched-out lesions were excluded. Prodromal symptoms and/or concomitant extraocular manifestations were recorded. Our analyses included the following parameters assessed at entry and at the 1 month-follow-up: best-corrected-visual-acuity (BCVA), results of slit-lamp examination, Humphrey visual field testing and fundus imaging by fluorescein angiography. OCT imaging was performed using the Stratus (Carl Zeiss Meditec, Jena, Germany) from 2002 until 2011, then with the Spectralis (Heidelberg Engineering Inc., Heidelberg, Germany) tomographers. The study was approved by the Ethics Committee of the French Society of Ophthalmology and adhered to the Declaration of Helsinki for research involving human subjects.

## Results

Out of 22 patients who had been diagnosed with APMPPE, 10 patients (1 man, 9 women) met our study criteria. The criteria for exclusion were atypical features in 3 cases: two with associated multifocal choroiditis and one with a systemic association of positive ANtineutrophil Cytoplasmic Antibodies (ANCAs). Seven cases were excluded for lack of follow-up and two treated patients were also excluded. Treatment in one of these cases was triggered by an episode of deafness with an onset 6 weeks after the ocular manifestations. In the second case, the treatment was triggered by an uncertain diagnosis with the possibility of Vogt-Koyanagi-Harada disease. Our patients’ demographic data and their extraocular manifestations are summarized in Table 1.

**Table 1:** Demographics, extra-ocular findings, BCVA at entry, and at 4 and 8 weeks

Patient #	Gender	Age	Prodromic symptoms (time interval prior to diagnosis)	Clinical signs of meningitis and CSF analysis		Initial BCVA (decimal)		BCVA at 4 weeks (decimal)		BCVA at 8 weeks (decimal)	
				Clinical signs	CSF analysis (cells/mm^3^)	RE	LE	RE	LE	RE	LE
1	F	20–30	Flu-like syndrome (1 week)	Headaches	Lymphocytic meningitis (121 cells)	HM	HM	0.8	0.6	1.0	0.9
2	F	20–30	Flu-like syndrome (3 weeks)	None	ND	FC	1.0	0.6	1.0	0.8	1.0
3	F	20–30	Gastro-enteritis and erythema nodosum (1 week)	None	Undetermined meningitis (11 cells)	1.2	1.2	0.9	1.0	1.0	1.0
4	F	10–20	None	None	ND	1.0	0.3	1.0	1.0	1.0	1.0
5	F	20–30	Flu-like syndrome	None	ND	0.9	0.8	0.9	0.8	1.0	1.0
6	M	20–30	Flu-like syndrome (1 week)	None	ND	0.8	0.25	1.0	0.8	1.0	0.8
7	F	10–20	None	None	ND	1.0	0.2	1.0	1.0	1.0	1.0
8	F	20–30	Fever and erythema nodosum (1 week)	None	ND	0.1	0.5	0.9	0.5	1.0	0.8
9	F	20–30	None	None	ND	1.0	0.8	1.0	1.0	1.0	1.0
10	F	30–40	Fever (10 days)	Headaches	ND	FC	1.0	1.0	1.0	NA	NA

*F* female, *M* Male, *BCVA* Best Corrected Visual Acuity, *RE* Right Eye, *LE* Left Eye, *ND* Not Done, *HM* Hand Motion vision, *FC* Finger Counting, *NA* Not Available

The mean patients’ age was 24.5 ± 4.2 years, with a female predominance: 9 women and 1 man. The mean follow-up was 11.45 ± 14.84 months with a median of 5.5 months.

Prodromal flu-like manifestations were reported in 7 of 10 patients before the onset of visual loss. The time interval between the prodromal manifestations and the observation of fundus lesions ranged from 7 days to 3 weeks. Out of the 10 patients, 4 had no work-up and 6 had the following investigations which were negative or normal: complete blood count, C-reactive protein dosage, interferon production assay, Purified Protein Derivative (PPD) test, syphilitic serology (TPHA-VDRL), ANCA, angiotensin converting enzyme dosage, as well as radiological exams: Computed Tomography of the brain and chest radiography. Two patients had a lumbar puncture revealing meningitis, with 121 cells/mm^3^ (100% lymphocytes) in one case and 11 cells/mm^3^ in the other. At 4 weeks, all extra-ocular symptoms had subsided in all of our patients.

**Fig. 1:**
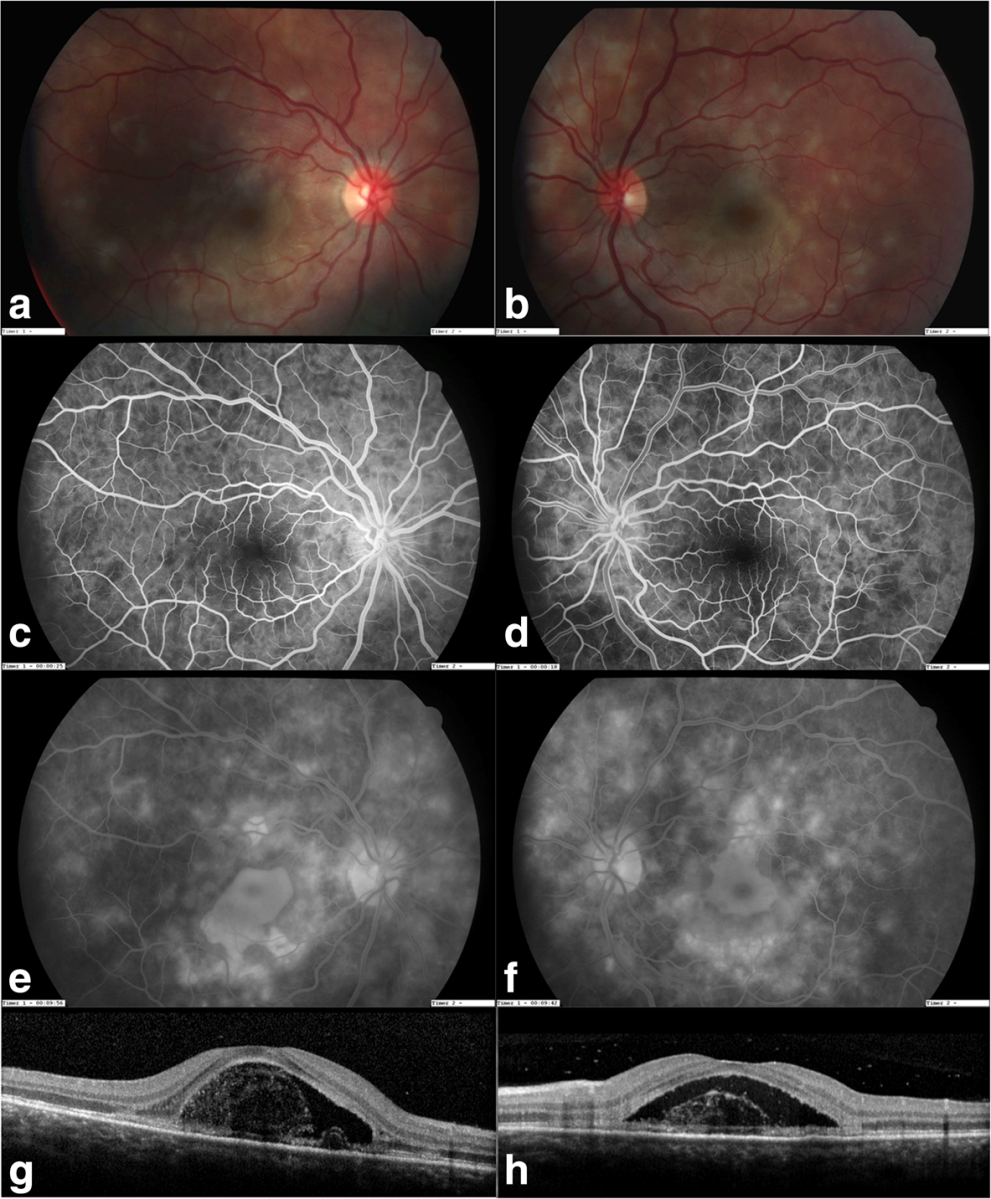
Ocular findings at presentation. **a**, **b**. Color photographs. **c**, **d**. Early stage of the fluorescein angiography with hypofluorescent lesions. **e**, **f**. Late phase of the fluorescein angiography with hyperfluorescent lesions. **g**, **h**. SD-OCT imaging: Sub-Retinal Fluid

**Fig. 2:**
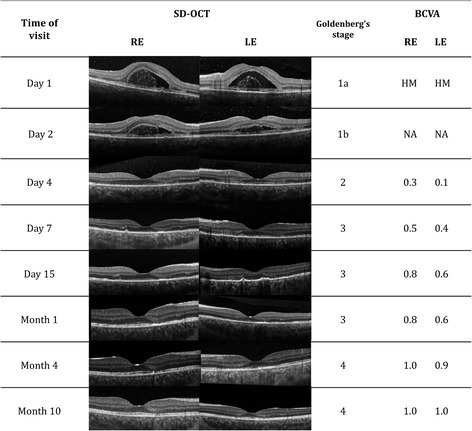
Follow-up of visual acuity, SD-OCT imaging and Goldenberg’s classification staging in a patient presenting with Sub-Retinal Fluid at entry

The mean LogMAR BCVA at presentation was 0.56 ± 0.81. Five of the 20 eyes had a decimal BCVA ≤0.1, while 11/20 eyes had a decimal BCVA ≥0.8 with a median of 0.8. Cells in the anterior chamber were observed in 9/20 eyes at onset and were < 2+ in all cases. The size of the whitish or yellowish placoid lesions ranged from 250 to 500 μm or more when merging, 13 eyes had more than 5 placoid lesions. The lesions were observed at the posterior pole in 19/20 eyes, as well as in the mid-periphery or in the periphery in 12/20 eyes. Figure 1 shows the imaging of a patient with a macular involvement. On OCT imaging, sub-retinal fluid was observed in 3 eyes, and alterations of the ellipsoid zone was seen in 7 eyes. At 4 weeks, the median decimal BCVA was 1.0 and the mean LogMAR acuity was 0.05 ± 0.089. At 8 weeks (9 patients), 16 eyes had a BCVA of 1.0, the median acuity was thus 1.0 and the mean acuity was 0.015 LogMar. Two eyes recovered a decimal BCVA of 1.0 after a follow-up longer than 8 weeks (at 10 months for one patient and at 5 months for the other one). The aspect of the lesions had evolved to a slight pigmentation at 4 weeks, except for 5 eyes for which the placoid lesions remained whitish-yellowish. The size of the lesions was unchanged or had decreased very moderately, their location was unchanged. On OCT, the sub-retinal fluid had disappeared in all affected eyes, however alterations of the ellipsoid zone remained in 3 eyes on OCT imaging, with an hyperreflectivity of the outer nuclear layer. Figure 2 presents the follow-up of a patient with SD-OCT and compares the visual acuity with SD-OCT imaging and staging using Goldenberg’s classification. At entry, subretinal fluid (SRF) was present, while acuity was limited to the detection of Hand Motion (HM). At 4 days, SRF had nearly disappeared and decimal BCVA improved to 0.3 OD and 0.1 OS. At 1 month, there was a persistent disruption of the ellipsoid zone and an hyperreflectivity of the Retinal Pigment Epithelium with a thinning of the Outer Nuclear Layer (ONL). BCVA had improved to decimal 0.8 and 0.6. At 4 months, BCVA was almost normal (OD:decimal 1.0 and OS: decimal 0.9). Finally, a complete recovery was observed at 10 months. OCT imaging showed a persistent thinning of the ONL and an irregularity of the ellipsoid zone, which might explain why some patients kept moderate visual consequences of their disease.

## Discussion

The number of patients in published series of cases of APMPPE is limited and ours is the fourth largest within the past decade and the largest with untreated patients [13, 17, 18]. As in other series, the majority of cases affected patients in their third decade [18–20]. The 9:1 female predominance observed in our series was greater than in other reports, but that may just be a chance finding due to the relatively small number of patients in our series. All prodromal syndromes observed in our series have also been previously reported. In other series the cumulated frequency of these various manifestations ranged from 18 to 61% and were most often estimated around 30% [17, 18, 21]. We did not identify any particular link between extra-ocular symptoms and ocular findings.

In spite of early reports of favourable outcomes in untreated APMPPE, subsequent studies have frequently comprised both treated and untreated patients [17, 18]. In the literature, the following triggers for treatment have been reported: macular localization of lesions, sub-retinal fluid on OCT and/or severe visual loss. The most commonly used treatment method were corticosteroids with different dosages, including occasionally initial IV methylprednisolone pulses. Two series of 11 and 21 untreated patients had also observed a good recovery in terms of BCVA with final decimal BCVA of 0.94 and 0.86 respectively [11, 12].

Overall our observation of the favorable recovery of the visual acuity was within the spectrum or better than that of other reports. The two most recent series with treated patients also showed a very good recovery of visual acuity with mean final decimal acuities of 0.9 and 0.67 [9, 17].

We cannot rule out that the following biases could have influenced our observations: 2 treated patients were excluded and 7 patients were lost to follow-up. Although three-fourth of the eyes in our study had macular lesions, a good visual recovery was nevertheless also observed in those cases. Initial severe visual loss, subretinal fluid or hyperpigmentation of plaques during the patients’ follow-up did not impede the recovery of a normal BCVA. Our study’s outcome measure was focused on BCVA but persistent visual field alterations after APMPPE have been reported in up to 67.9% of patients [22]. Some patients may complain of an imperfect vision in the aftermath of their attack, in spite of a normal BCVA. In these cases, central visual field testing or microperimetry can be useful to identify visual defects at the site of healed atrophic or hyperpigmented plaques. In our study, the results of visual field testing was only available in 5 patients (data not shown). We cannot rule out treatment could favorably influence the outcome of visual field testing in subgroups of patients after their attack of APMPPE.

A classification of the OCT findings observed in APMPPE was suggested by Goldenberg et al. [9]. This included a first stage with sub-retinal fluid and later stages with outer nuclear layer (ONL) hyperreflectivity, thinning of the outer nuclear layer, disruption of the ellipsoid zone, hyperreflective bands of ellipsoid and RPE. Future studies entirely based on SD-OCT will allow to better understand the relation between BCVA and stages of APMPPE according to Goldenberg’s classification. In some cases, the early stage of APMPPE may share features with the early stage of Vogt-Kayanagi-Harada disease [23]. Extraocular features reported in association with APMPPE have included neurological manifestations, yet in our series, headaches and/or lymphocytic meningitis were not a trigger for initiating treatment [24]. A case was reported, for which corticosteroids at a dosage of 80 mg per day did not prevent a stroke due to cerebral vasculitis [17].

## Conclusions

Our series confirms that untreated patients with APMPPE can have a favourable outcome. Whether this applies to all cases of the disease remains unknown and there is no consensus regarding the factors that should trigger treatment in selected patients. As APMPPE is a rare disease, the assessment of the benefits of treatment would probably require an international trial to recruit enough patients for the gathering of evidence-based data. Until then, decisions to treat patients with APMPPE or not will remain rather empirical.

## Abbreviations

ANCAs: ANtineutrophil cytoplasmic antibodies
APMPPE: Acute posterior multifocal placoid pigment epitheliopathy
BCVA: Best-corrected-visual-acuity
CNS: Central nervous system
IS: Inner segment
OCT: Optical coherence tomography
ONL: Outer nuclear layer
OS: Outer segment
PPD: Purified protein derivative
RPE: Retinal pigment epithelium
SD-OCT: Spectral domain optical coherence tomography
SRF: Sub-retinal-fluid

## References

[1] Deutman AF, Oosterhuis JA, Boen-Tan TN, Aan de Kerk AL (1972). Acute posterior multifocal placoid pigment epitheliopathy. Pigment epitheliopathy of choriocapillaritis?. Br J Ophthalmol.

[2] Chiquet C, Lumbroso L, Denis P, Papo T, Durieu I, Lehoang P (1999). Acute posterior multifocal placoid pigment epitheliopathy associated with Wegener's granulomatosis. Retina.

[3] Dolz-Marco R, Sarraf D, Giovinazzo V, Freund KB. Optical coherence tomography angiography shows inner choroidal ischemia in acute posterior multifocal placoid pigment epitheliopathy. Retin Cases Brief Rep. 2016;11(Suppl 1):S136–S143. 10.1097/ICB.0000000000000473 27759710

[4] Salvatore S, Steeples LR, Ross AH, Bailey C, Lee RW, Carreno E (2016). Multimodal imaging in acute posterior multifocal Placoid pigment Epitheliopathy demonstrating obstruction of the Choriocapillaris. Ophthalmic Surg Lasers Imaging Retina.

[5] Jones NP (1995). Acute posterior multifocal placoid pigment epitheliopathy. Br J Ophthalmol.

[6] Quillen DA, Davis JB, Gottlieb JL, Blodi BA, Callanan DG, Chang TS, Equi RA (2004). The white dot syndromes. Am J Ophthalmol.

[7] O'Halloran HS, Berger JR, Lee WB, Robertson DM, Giovannini JA, Krohel GB, Meckler RJ, Selhorst JB, Lee AG, Nicolle DA, O'Day J (2001). Acute multifocal placoid pigment epitheliopathy and central nervous system involvement: nine new cases and a review of the literature. Ophthalmology.

[8] Brezin AP, Massin-Korobelnik P, Boudin M, Gaudric A, LeHoang P (1995). Acute posterior multifocal placoid pigment epitheliopathy after hepatitis B vaccine. Arch Ophthalmol.

[9] Goldenberg D, Habot-Wilner Z, Loewenstein A, Goldstein M (2012). Spectral domain optical coherence tomography classification of acute posterior multifocal placoid pigment epitheliopathy. Retina.

[10] Birnbaum AD, Blair MP, Tessler HH, Goldstein DA (2010). Subretinal fluid in acute posterior multifocal placoid pigment epitheliopathy. Retina.

[11] Williams DF, Mieler WF (1989). Long-term follow-up of acute multifocal posterior placoid pigment epitheliopathy. Br J Ophthalmol.

[12] Vianna R, van Egmond J, Priem H, Kestelyn P (1993). Natural history and visual outcome in patients with APMPPE. Bull Soc Belge Ophtalmol.

[13] Fiore T, Iaccheri B, Androudi S, Papadaki TG, Anzaar F, Brazitikos P, D'Amico DJ, Foster CS (2009). Acute posterior multifocal placoid pigment epitheliopathy: outcome and visual prognosis. Retina.

[14] Saraux H, Pelosse B (1987). Acute posterior multifocal placoid pigment epitheliopathy. A long-term follow-up. Ophthalmologica.

[15] Taich A, Johnson MW (2008). A syndrome resembling acute posterior multifocal placoid pigment epitheliopathy in older adults. Trans Am Ophthalmol Soc.

[16] Grkovic D, Oros A, Bedov T, Karadzic J, Gvozdenovic L, Jovanovic S (2013). Acute posterior multifocal placoid pigment epitheliopathy-retinal “white dot syndrome”. Med Glas.

[17] Thomas BC, Jacobi C, Korporal M, Becker MD, Wildemann B, Mackensen F (2012). Ocular outcome and frequency of neurological manifestations in patients with acute posterior multifocal placoid pigment epitheliopathy (APMPPE). J Ophthalmic Inflamm Infect.

[18] Bures-Jelstrup A, Adan A, Casaroli-Marano R (2007). Acute posterior multifocal placoid pigment epitheliopathy. Study of 16 cases. Arch Soc Esp Oftalmol.

[19] Roberts TV, Mitchell P (1997). Acute posterior multifocal placoid pigment epitheliopathy: a long-term study. Aust N Z J Ophthalmol.

[20] Taich A, Johnson MW (2009). A syndrome resembling acute posterior multifocal placoid pigment epitheliopathy in older adults. Retina.

[21] Crawford CM, Igboeli O (2013). A review of the inflammatory chorioretinopathies: the white dot syndromes. ISRN Inflamm.

[22] Wolf MD, Alward WL, Folk JC (1991). Long-term visual function in acute posterior multifocal placoid pigment epitheliopathy. Arch Ophthalmol.

[23] Li B, Bentham RJ, Gonder JR (2016). A case of unilateral and spontaneously resolving posterior uveitis with overlapping features of Vogt-Koyanagi-Harada disease and acute posterior multifocal placoid pigment epitheliopathy. Spring.

[24] Algahtani H, Alkhotani A, Shirah B (2016). Neurological manifestations of acute posterior multifocal placoid pigment epitheliopathy. J Clin Neurol.

